# Comparative Study of Accommodative Function and Binocular Vision in Patients With Primary Angle‐Closure Disease

**DOI:** 10.1002/kjm2.70134

**Published:** 2025-10-31

**Authors:** Feng‐Rui Yang, Zhi‐Qiao Liang, Ye Lu, Yao Ma, Kun Lv, Qian Zhang, Hui‐Juan Wu

**Affiliations:** ^1^ Department of Ophthalmology Peking University People's Hospital Beijing China; ^2^ Eye Diseases and Optometry Institute, Beijing Key Laboratory of Diagnosis and Therapy of Retinal and Choroid Diseases, College of Optometry Peking University Health Science Center Beijing China

**Keywords:** accommodative facility, amplitude of accommodation, binocular vision, primary angle‐closure diseases, primary angle‐closure glaucoma

## Abstract

The age‐related decline in accommodative function after the age of 50 years corresponds with an increasing incidence of primary angle‐closure disease (PACD); however, the interaction between this decline and PACD remains unexamined. Additionally, refractive error‐accommodation associations in elderly individuals, which are critical for PACD pathophysiology, remain unclear, despite a prior pediatric focus. This study evaluated visual functions (including binocular vision, vergence function, and accommodation function) in patients diagnosed with PACD. A total of 51 patients (102 eyes) were included in this prospective study. The subjects were categorized into the following three groups: primary angle‐closure/primary angle‐closure glaucoma (PAC/PACG), primary angle‐closure suspect (PACS), and a control group. Various parameters, including best‐corrected near visual acuity, refractive error, Worth's 4 Dot test results, stereoacuity, vergence facility, accommodative facility (AF), and amplitude of accommodation (AMP), were measured and compared across the groups. A negative correlation was noted between AMP and refractive error (*p* < 0.001). After controlling for age and refractive error, no significant difference in AMP was detected; however, a statistically significant difference in AF was noted among the groups. PACS (*p* = 0.002) and PAC/PACG (*p* = 0.048) emerged as independent predictors of strong AF compared with normal controls. No significant differences in best‐corrected near visual acuity, binocular vision, or vergence facility were observed between the groups. This research demonstrated that patients diagnosed with PACS and PAC/PACG exhibited stronger AF compared with the control group. Furthermore, a negative correlation between AMP and refractive error was observed within the elderly population. This research characterizes the correlation between refractive status and accommodative function, while establishing a link between accommodative function and PACD. Future studies on PACD pathogenesis and clinical management should prioritize accommodative function as a key research focus.

## 1 Introduction

Primary angle‐closure disease (PACD) encompasses a spectrum of conditions that range from a narrow anterior chamber angle to advanced glaucoma. The International Society for Geographical and Epidemiological Ophthalmology classifies PACD into the following three categories: primary angle‐closure suspect (PACS), primary angle‐closure (PAC), and primary angle‐closure glaucoma (PACG) [1]. Although these conditions differ in severity, they share a common pathophysiological foundation, and progression from one pathophysiological stage to another is possible. PACS is identified as the initial stage of angle closure, which is characterized by the presence of multiple quadrants of angle closure (as determined via gonioscopy) [1]. The progression from PACS to PAC occurs when peripheral anterior synechiae or elevated intraocular pressure is elicited [2]. PACG is defined as a PAC eye that exhibits signs of glaucomatous optic nerve damage, which may include an abnormal cup‐to‐disc ratio or glaucomatous visual field defects [3]. Notably, Asia demonstrates the highest global incidence of PACD [4, 5]. In China, the overall prevalence of glaucoma is estimated to be 2.58%, with PACG representing the predominant form, which accounts for 54.42% of the cases [6]. The increasing prevalence of PACG in China highlights the urgent need for improved diagnostic and therapeutic strategies.

PACS, PAC, and PACG represent a progressive continuum of disease [7]. Although the established definitions of these three conditions have clarified the associated structural damage, the differences in visual function among these conditions have not been thoroughly examined. Additionally, the mechanisms that facilitate the progression from PACS to PACG remain unclear. In contrast to the abnormal cup‐to‐disc ratio and glaucomatous visual field defects, which represent outcomes of elevated intraocular pressure [8], knowledge of the etiological factors leading to angle closure and increased intraocular pressure is critical for disease prevention and the maintenance of vision. It has been suggested that further evaluations of visual function (such as contrast sensitivity, color vision, and electroretinography evaluations) may yield additional insights when considered in conjunction with visual field assessments [9]. The primary factors contributing to angle closure and elevated intraocular pressure in patients with PACD are located in the anterior segment of the eye; therefore, the evaluation of visual function (particularly accommodative function) is highly important. Meanwhile, visual field defects predominantly occur in the peripheral regions in the early stage of glaucoma. In accommodative control, the central visual field plays a dominant role by providing real‐time feedback on optical clarity during fixation, thereby driving refractive adjustments. Consequently, intact central visual field function is essential for normative accommodative responses. Therefore, early‐stage glaucoma patients are less likely to experience impairments in accommodation function attributed to their condition [10, 11].

In clinical practice, adequate correction of presbyopia may lead to the deepening of the anterior chamber in patients diagnosed with PACD. Although this phenomenon has yet to be substantiated by large‐scale studies, it may suggest a potential relationship between accommodative function and the onset and progression of PACD. It is well documented that individuals with hyperopia are more susceptible to PACD [12, 13]. Consequently, the impact of ametropia on accommodative function is a significant concern. Research has indicated that the amplitude of accommodation (AMP) is positively correlated with refractive errors [14], whereas accommodative facility (AF) is negatively correlated with refractive errors [15]. However, previous investigations on this topic have predominantly focused on pediatric and young adult populations, thus leading to a gap in the understanding of this topic in middle‐aged and elderly cohorts. Therefore, further research on this topic in these cohorts is warranted.

Research has indicated that primary open angle glaucoma is linked to a deterioration in binocular vision [16] and that this decline in binocular vision is correlated with the thinning of the retinal nerve fiber layer (RNFL) [17]. However, it remains unknown whether other factors influence binocular vision in addition to RNFL thinning and visual field defects. This study involved patients with PACD and normal subjects (which ensured that there were no significant differences in visual field defects) to investigate the relationship between binocular vision and PACD.

To address the aforementioned questions related to this topic, we proposed several hypotheses: (1) in the elderly population (aged 50 years and older), AMP is positively correlated with refractive errors, whereas AF is negatively correlated with refractive errors; (2) there are significant differences in AMP and AF among patients with PAC/PACG, patients with PACS, and individuals with normal ocular health; and (3) binocular vision significantly varies among PAC/PACG patients, PACS patients, and normal subjects, particularly in patients without obvious visual field defects.

To evaluate the aforementioned hypotheses, this study constitutes the first systematic investigation into the differences in visual function assessment results among patients diagnosed with PACD and normal subjects. The visual function assessments conducted in this research included monocular and binocular best‐corrected near visual acuity (BCNVA), the Worth's 4 Dot (W4D) test, stereoacuity, vergence facility (VF), AF, and AMP. The principal objective of this study was to examine the effects of PACD on various aspects of human visual function.

## 2 Material and Methods

### 2.1 Patients

This prospective study was approved by the ethics committee of the affiliated hospital and adhered to the tenets of the Declaration of Helsinki. Written informed consent was obtained from all of the participants. Between January 2022 and August 2024, a total of 51 patients (102 eyes) were recruited from the ophthalmology department of a university‐affiliated hospital. The cohort comprised 16 patients with PACS, 18 patients with PAC/PACG, and 17 control individuals. The control group included individuals who were healthy, as well as those diagnosed with presbyopia, asthenopia, or vitreous opacity.

The diagnostic criteria for PACD were established by the International Society for Geographical and Epidemiological Ophthalmology [1]. PACS is characterized by the inability to visualize more than 270° of the posterior trabecular meshwork via gonioscopy in the absence of elevated intraocular pressure, peripheral anterior synechiae, or glaucomatous optic nerve defect. The diagnosis of PACG necessitates the presence of trabecular obstruction caused by the peripheral iris, which may manifest as PAS, elevated IOP, iris whorling, “glaucomflecken” lens opacities, or significant pigment deposition on the trabecular surface, in addition to demonstrable glaucomatous optic nerve damage, as indicated by an abnormal cup‐to‐disc ratio or glaucomatous visual field defects. All of the diagnoses were conducted by a single experienced glaucoma specialist.

Participants were recruited for the study based on the following baseline criteria: (1) aged between 50 and 90 years; (2) had a confirmed diagnosis of PAC/PACG or PACS, with the exception of those in the control group; and (3) had a BCNVA exceeding 0.05 in both monocular and binocular assessments. The exclusion criteria were as follows: (1) individuals with intraocular lens implantations; (2) individuals presenting with secondary angle closure conditions, such as lens dislocation or neovascular glaucoma; (3) individuals with a history of ocular diseases, including ocular trauma, uveitis, or proliferative diabetic retinopathy; (4) individuals with difficulties in following instructions, such as those with dementia or hearing impairments; and (5) individuals who declined to participate in the visual function examination.

### 2.2 Examination

Baseline characteristics and medical histories were systematically collected for all of the participants in this study. Each patient underwent a thorough visual function assessment utilizing an intelligent visual function evaluation instrument (Youyan Technology Co. Ltd., Wenzhou, Zhejiang, China) [18]. The outcomes generated by this instrument included BCNVA, VF, AF, AMP, W4D testing, and stereoacuity. All of the required optotypes for the test were displayed on a screen. The patient's head was fixed, and the screen was viewed through two holes in front of both eyes. Different lenses could be placed in the two viewing holes, and the distance between the screen and the eyes could be adjusted. In the BCNVA test, the optotypes of the same size as the standard logarithmic near vision chart were displayed in descending order on the screen located 40 cm away from the eyes. VF and AF were detected in a manner similar to that of a flipper, and cycles per minute were recorded. The instrument could switch between +2.00 D and −2.00 D lenses in the AF test and 3∆ base‐in and 12∆ base‐out in the VF test. The AMP test used a combination of the minus lens method and push‐up method. Additionally, the W4D and stereoacuity examinations were performed when the refractive error had been completely corrected. The green and red filters were placed in front of the left and right eyes, respectively. The W4D and random dot stereograms were displayed on the screen, and the observations reported by the patients were recorded.

Patients in the control group received fundus examinations by an experienced glaucoma doctor to ensure that they had no glaucoma optic disc damage. Patients in the PACS and PAC/PACG groups underwent RNFL thickness tests (Spectralis OCT, Heidelberg Engineering Inc., Heidelberg, Germany), visual field perimetry tests (Swedish Interactive Threshold Algorithm [SITA] 24‐2 test of the Humphrey visual field analyzer 750i, Carl Zeiss Meditec, Dublin, CA), and ultrasound biomicroscopy (UBM) tests (Aviso, Quantel Medical Inc., Bozeman, MT, USA). Furthermore, all of the patients received manifest refraction measurements conducted with an autorefractor (ARK‐700A, NIDEK, Japan).

### 2.3 Statistical Analysis

The data are presented as the means and changes from baseline ± standard error. The Kolmogorov–Smirnov test was employed to assess the distribution of the data. When comparing continuous variables among the three groups, analysis of variance was conducted for variables with homogeneity of variance, whereas the Kruskal–Wallis test was utilized when variances were nonhomogeneous. A linear mixed‐effects model (LMM) was used when comparing data from both eyes [19], such as AMP, AF, and refractive error results. Chi‐square tests were performed to compare binocular categorical variables across the groups. A *p* value of less than 0.05 was considered to be statistically significant.

## 3 Results

### 3.1 Demographic Information and Baseline Data

The demographic information and baseline data of the study participants are detailed in Table 1. The study included a total of 51 patients (102 eyes). No statistically significant differences were identified in terms of sex, age, BCNVA, or visual field defects among the three groups. Furthermore, a statistically significant difference in refractive errors was noted among the three groups (*p* = 0.003, LMM). In the pairwise comparisons, the control group (*p* = 0.004) and the PAC/PACG group (*p* = 0.035) demonstrated a greater prevalence of myopia compared to the PACS group, whereas no statistically significant difference was observed between the control and PAC/PACG groups (*p* = 0.054).

**TABLE 1:** Demographic, visual acuity and lens status data of the three groups.

Variables	Control	PACS	PAC/PACG	*p*
Number of patients	17	16	18	
Number of eyes	34	32	36	
Age	63.18 ± 8.12 (50–77)	66.44 ± 9.13 (50–78)	64.67 ± 6.72 (52–76)	0.509
Gender (Male:Female)	5:12	3:13	6:12	0.677
BCNVA	0.33 ± 0.16	0.42 ± 0.23	0.41 ± 0.16	0.546
Visual field defect (MD)	—	−2.94 ± 3.31	−3.51 ± 4.40	0.754
Refractive errors (D)	−0.88 ± 3.31	1.96 ± 1.68	0.81 ± 1.38	**0.003**

*Note*: All of the values are presented as the means ± standard deviations, unless stated otherwise. Bold values indicates statistically significant *p* < 0.05.

Abbreviations: BCNVA, best corrected near visual acuity; MD, mean deviation; PAC, primary angle‐closure; PACG, primary angle‐closure glaucoma; PACS, primary angle‐closure suspect.

**FIGURE 1:**
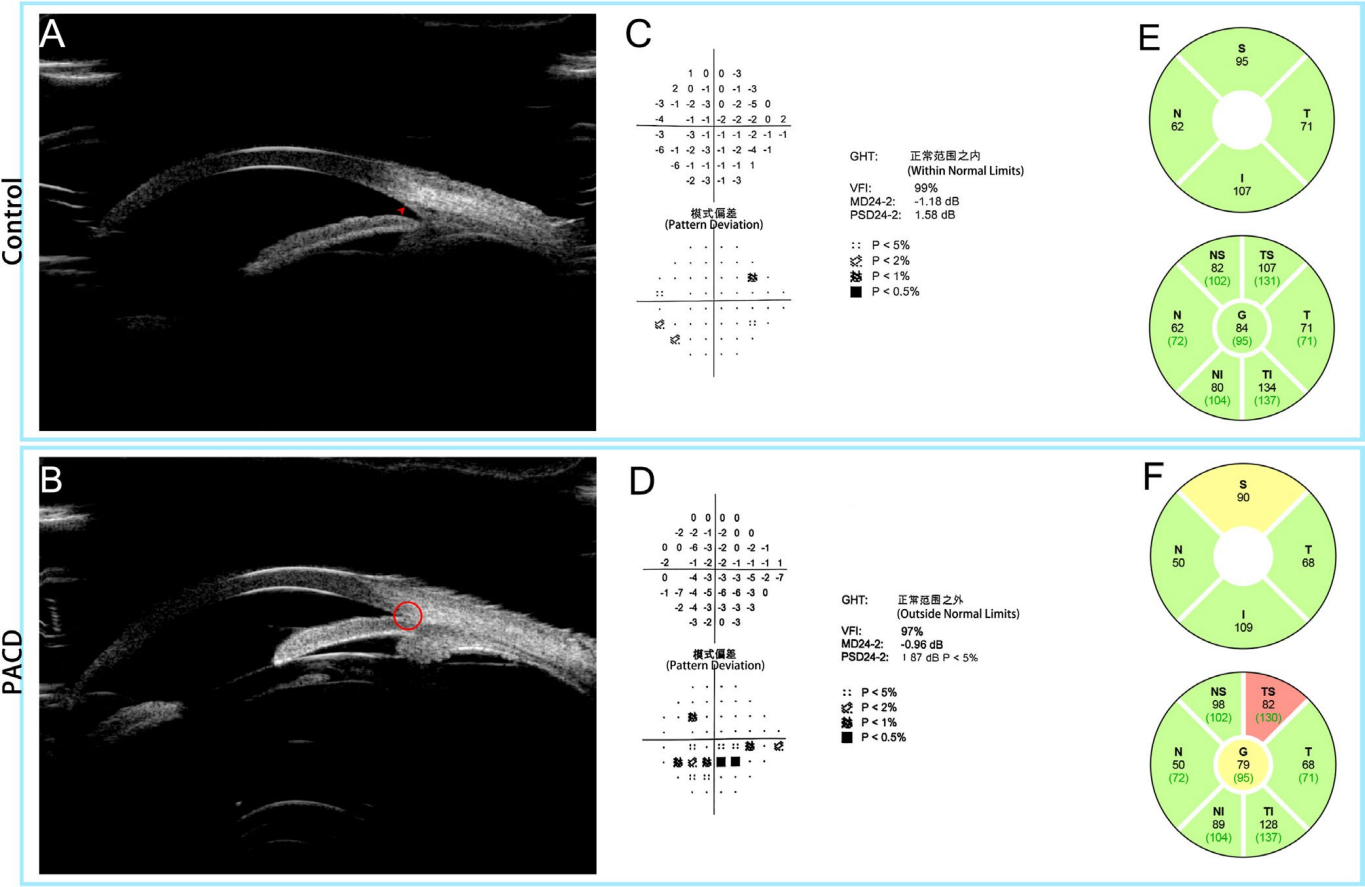
Typical examination findings in patients from PAC/PACG group and control group. (A) UBM imaging in the control group revealed open angles with clearly visible scleral spurs (red arrowhead). (B) In patients diagnosed with PACG, the UBM images revealed closure of the anterior chamber angle (red circle), an increase in iris thickness, and anterior displacement of the ciliary body. (C and D) In the pattern deviation map of the visual field test, artifacts such as media opacities are corrected. Values represent the deviation in light sensitivity from age‐matched normal values. Grayscale represents the probability of light sensitivity abnormality. (E and F) In RNFL thickness test, black numbers indicate average RNFL thickness (μm) in the region, while green numbers denote the demographic‐matched average. The color block: Green, within normal limits (> 5%); yellow, borderline (< 5%); red, outside normal limits (< 1%). Abbreviations: G, global; GHT, glaucoma hemifield test; I, inferior; MD, mean deviation; N, nasal; NI, nasal‐inferior; NS, nasal‐superior; PSD, pattern standard deviation; S, superior; T, temporal; TI, temporal‐inferior; TS, temporal‐superior; VFI, visual field index.

Figure 1 illustrates the characteristic findings from UBM (Figure 1A,B), visual field test (Figure 1C,D), and RNFL thickness assessments (Figure 1E,F) in control individuals and patients diagnosed with PACG, respectively. UBM images of PACG patients revealed angle closure and anterior displacement of the ciliary body as compared with the control group (Figure 1A,B). Both visual field and RNFL thickness in the control group were within normal limits (Figure 1C,E). The visual field test reveals an inferior arcuate scotoma (Figure 1D), with reduced RNFL thickness in the temporal‐superior (TS) quadrant (Figure 1F). This finding conforms to the diagonal correspondence of RNFL and visual field damage in glaucoma [20, 21]. However, the visual field defects were at an early stage and did not affect accommodation function.

### 3.2 Impact of Ametropia on Accommodative Function

**FIGURE 2:**
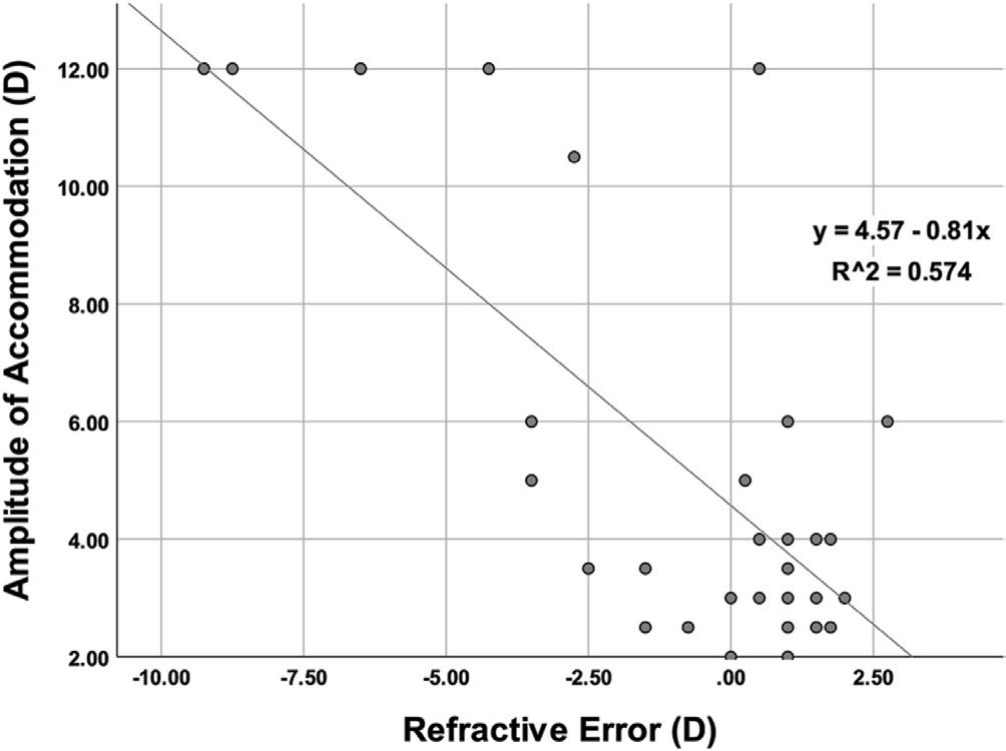
Correlation between AMP and refractive errors. A statistically significant correlation was observed (*p* < 0.001, *R* ^2^ = 0.574).

To mitigate the potential impact of the PACD on accommodative function, normal subjects were included in the analysis. All of the variables deemed to be relevant to AMP were included in the LMM. As illustrated in Table 2, the refractive error exhibited a negative predictive relationship with AMP, thereby indicating that a greater propensity toward hyperopia was correlated with diminished AMP. Similar results were noted across all of the participants in this study, encompassing individuals who were diagnosed with PACD. The relationship between the refractive error and AMP is depicted in Figure 2.

**TABLE 2:** Linear mixed‐effects model for factors potentially correlated with AMP.

Variable	Estimate (95% CI)	Standard error	*t*	*p*
Intercept	3.150 (−7.055, 13.355)	4.727	0.666	0.517
Age	0.027 (−0.140, 0.194)	0.077	0.350	0.732
Gender	−1.019 (−3.974, 1.937)	1.370	−0.743	0.470
Refractive error	−0.820 (−1.152, −0.489)	0.156	−5.252	**< 0.001**

*Note*: Bold values indicates statistically significant *p* < 0.05.

To examine the association between AF and ametropia, we conducted an analysis of AF that closely mirrored the methodology employed for assessing AMP. In contrast to the findings related to AMP, the AF results indicated that the refractive error did not serve as a reliable predictor of AF in individuals lacking any underlying abnormalities, as demonstrated in Table 3.

**TABLE 3:** Linear mixed‐effects model for factors potentially correlated with AF.

Variable	Estimate (95% CI)	Standard error	*t*	*p*
Intercept	6.362 (−2.995, 15.718)	4.343	1.465	0.166
Age	−0.081 (−0.234, 0.073)	0.071	−1.136	0.276
Gender	0.289 (−2.417, 2.995)	1.257	0.230	0.822
Refractive error	0.067 (−0.226, 0.359)	0.139	0.478	0.638

### 3.3 Comparative Analysis of Accommodative Function Across the Different Groups

**FIGURE 3:**
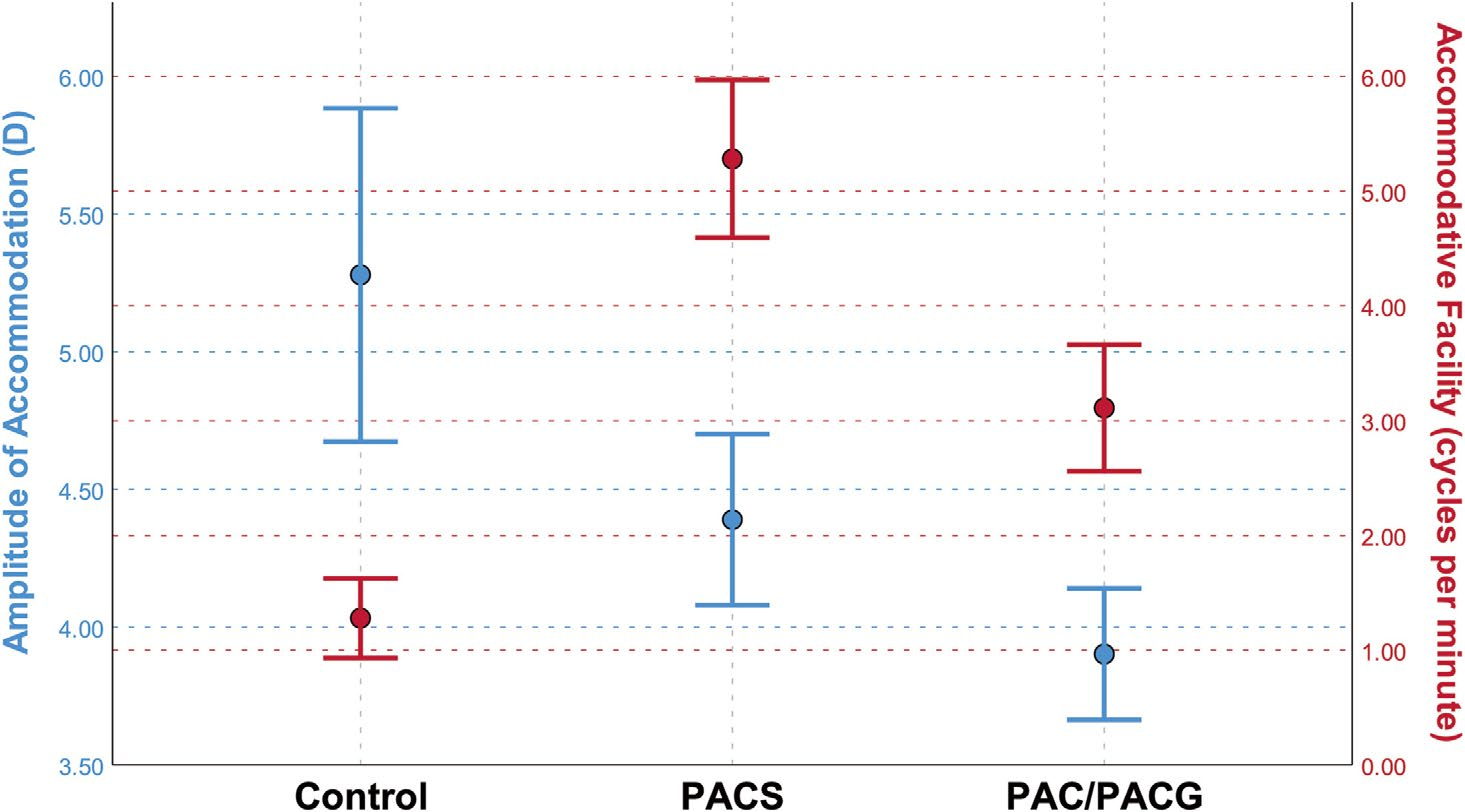
AMP and AF of the control, PACS, and PAC/PACG groups. The midpoint of the error bars represents the mean, and the length of the error bars indicates the standard error.

The mean values and standard errors of AF and AMP in the control, PACS, and PAC/PACG groups are shown in Figure 3, from which lower AMP and higher AF can be observed in patients with PACD. However, it is important to consider that various factors, including refractive errors and age, can influence accommodative function, along with the fact that refractive errors can vary significantly across different groups. Therefore, these variables must be incorporated into the model to accurately assess the differences in accommodative function among distinct groups. In our analysis, we included age, sex, refractive error, and diagnosis in the LMM and conducted comparisons of AMP and AF between pairs of groups. As presented in Table 4, after adjusting for the effects of varying refractive statuses, the AF in the PACS group (*p* = 0.002) and the PAC/PACG group (*p* = 0.048) remained significantly greater than that in the control group, thereby suggesting a potential association between PACD and increased AF. Conversely, no significant differences in AMP were detected among the groups.

**TABLE 4:** Comparison results between the AMP and AF groups, with age, sex, and refractive error included in the LMM.

Variables		Estimate (95% CI)	Standard error	*t*	*p*
AMP	Control—PACS	−0.556 (−2.328, 1.215)	0.866	−0.642	0.526
	Control—PAC/PACG	0.220 (−1.032, 1.472)	0.613	0.359	0.722
	PACS—PAC/PACG	0.081 (−0.867, 1.029)	0.464	0.175	0.862
AF	Control—PACS	−3.927 (−6.273, −1.580)	1.149	−3.416	**0.002**
	Control—PAC/PACG	−1.671 (−3.324, −0.017)	0.811	−2.061	**0.048**
	PACS—PAC/PACG	1.996 (−0.427, 4.419)	1.187	1.284	0.103

*Note*: All of the values are presented as the means ± standard deviations, unless stated otherwise. Bold values indicates statistically significant *p* < 0.05.

Abbreviations: AF, accommodative facility; AMP, amplitude of accommodation; PAC, primary angle‐closure; PACG, primary angle‐closure glaucoma; PACS, primary angle‐closure suspect.

### 3.4 Binocular Vision and Vergence Function

The findings from the W4D, stereoacuity, and VF assessments are presented in Table 5. The analysis revealed no statistically significant differences among the three groups in the W4D test. Given that the lowest measurement value for stereoacuity on the instrument is 800″, the results were categorized into two groups, and a chi‐square test was conducted. The analysis indicated that there were no statistically significant differences in stereoacuity among the three groups. The analysis also revealed no statistically significant differences in the VF test results between the control group, the PACS group, and the PAC/PACG groups.

**TABLE 5:** Comparison of the results of the Worth's 4 Dot test, stereoacuity test, and vergence facility test between the control group, PACS group, and PAC/PACG group.

		Control	PACS	PAC/PACG	*p*
W4D	Fusion	15 (88%)	15 (94%)	15 (83%)	0.861
	Suppression	2 (12%)	1 (6%)	3 (17%)	
Stereoacuity	≤ 800″	9 (53%)	6 (38%)	6 (33%)	0.522
	> 800″	8 (47%)	10 (62%)	12 (67%)	
VF (cycles per minute)		7.74 ± 4.35	5.56 ± 4.40	4.92 ± 4.94	0.178

*Note*: W4D and stereoacuity values are presented as numbers (%).

Abbreviations: PAC, primary angle‐closure; PACG, primary angle‐closure glaucoma; PACS, primary angle‐closure suspect; VF, vergence facility; W4D, Worth's 4 Dot.

## 4 Discussion

In this study, we conducted a comparative analysis of visual function examination outcomes among PAC/PACG patients, PACS patients, and normal controls. In terms of accommodative function, both PACS and PAC/PACG patients exhibited greater AF than normal subjects. Furthermore, in normal subjects, AMP exhibited statistically significant correlations with refractive errors. This research aims to resolve a significant gap in the current literature based on several notable strengths. First, this study represents the first systematic comparison of accommodative function between patients with various pathological stages of PACD and normal controls. Second, our study introduces examinations of the correlation between refractive errors and accommodative parameters in individuals aged over 50 years, thereby extending the scope of previous investigations that have predominantly focused on pediatric and young adult populations.

Previous research has predominately focused on the relationships among AMP, AF, and refractive errors in pediatric and young adult populations. Radhakrishnan et al. reported lower AF in myopic individuals compared to emmetropic individuals within the age range of 20–32 years [22], whereas Pandian et al. reported similar findings in a younger cohort aged 6–7 years [15]. Conversely, Maheshwari et al. reported higher AMP in myopic individuals than in emmetropic individuals among participants aged 11–30 years [23]. A meta‐analysis conducted by Bernal‐Molina et al. corroborated this trend regarding AMP and provided a formula that elucidates the relationship [14]. Collectively, these studies have established a positive correlation between AF and refractive error, as well as a negative correlation between AMP and refractive error in children and young adults. However, there is a notable absence of research addressing the correlation between accommodation and refractive error in individuals aged 50 years and older. In this investigation, we established a negative correlation between AMP and refractive error in individuals older than 50 years of age. Further research with larger sample sizes may be necessary to validate these findings. In contrast to younger populations, no positive correlation was observed between AF and refractive error in older adults. This phenomenon may be attributed to factors such as lens hardening, which results in a reduction in and convergence of AF within the elderly population.

AF denotes the rate at which individuals respond to variations in accommodative stimuli. To date, no research has established a direct correlation between diminished AF and the pathogenesis or progression of glaucoma. In the present study, we identified a statistically significant disparity in AF among the PAC/PACG, PACS, and control groups. After adjusting for age and refractive error, PACD served as an independent predictor of greater AF. The observed differences in AF may be attributed to variations in ciliary muscle contractility. Enhanced ciliary muscle contractility (coupled with persistent regulatory spasms) may facilitate anterior movement of the ciliary body, thereby compressing the iris root and narrowing the anterior chamber angle. It has been reported that Chinese individuals have significantly smaller anterior chamber areas and anterior chamber volumes compared with Caucasian individuals [24]. Moreover, a thinner ciliary body and more anteriorly rotated ciliary processes have also been reported in Chinese individuals [25]. These characteristics may increase the susceptibility of Chinese patients with shallow anterior chambers to enhanced ciliary muscle contractility, thus resulting in PACD. However, a study from Germany evaluated enucleated human eyes and revealed that eyes with an open anterior chamber angle exhibited greater ciliary muscle size and increased ciliary body stroma compared to those eyes with a closed angle [26], which may contradict our hypothesis. This discrepancy was attributed to a reduction in the unconventional outflow of aqueous humor, thus potentially resulting in a “drying‐up” of the ciliary body stroma. Differences in eye structures between different ethnicities may explain this contradiction.

Certain theories suggest that the pathogenesis of primary open‐angle glaucoma and normal‐tension glaucoma may be associated with “sustained stress on the ocular accommodative mechanism” [27]. Additionally, some studies have indicated that presbyopia and glaucoma may share similar pathophysiological mechanisms. Kaufman et al. suggested that each accommodative effort could exert a peripheral “pull” on the optic nerve via the ciliary muscle and choroid, which may contribute to glaucomatous optic nerve defects [28]. Nevertheless, no existing theory has adequately elucidated the relationship between AMP and PACG. Future research may focus on assessing the anterior chamber depth and angle in patients with PACD under varying accommodative conditions.

Binocular vision is a fundamental component of human visual function, encompassing the capacity to align the eyes in the motor aspect and integrate their images to perceive a dynamic three‐dimensional environment. The three dimensions of binocular vision (ranging from basic to more complex dimensions) include simultaneous vision, fusion, and stereopsis [29]. Research has indicated a potential association between glaucoma and impaired binocular vision. For example, Park et al. [14] reported that patients with primary open‐angle glaucoma exhibited diminished stereoacuity compared with control subjects. One hypothesis posits that the thickness of the RNFL may impact stereopsis. Alternatively, variations in the extent of binocular damage could contribute to a reduction in stereoacuity. For example, Dhar et al. reported that individuals with reduced RNFL thickness demonstrated lower stereoacuity for both distant and near vision [17]. Additionally, stereoacuity has been linked to defects in the central visual field [30] and tends to deteriorate as glaucoma progresses [17, 31]. In the present study, we observed no difference in binocular vision among the three study groups or in the pairwise comparisons. This result may be due to the fact that the PACG patients included in this study demonstrated no statistically significant visual field defects, which is consistent with the hypotheses of previous studies proposing that binocular vision impairment is related to reduced RNFL thickness and central visual field defects. However, few studies have examined binocular vision as a component of functional impairments in PACG. Consequently, further investigations are warranted to elucidate the role of binocular vision in PACG.

Several limitations of this study should be noted. First, the limited sample size may impact the generalizability of our findings. Additionally, the visual function examination was not comprehensive. Assessments such as negative relative accommodation, positive relative accommodation, and color vision testing could provide more extensive information. Moreover, the systematic collection of visual field data and RNFL thickness measurements may further help in elucidating the mechanisms underlying the observed visual function results. Furthermore, a focus on these limitations in future studies could strengthen the understanding of visual function in patients with PACD.

In this study, we performed a systematic comparison of visual function examination outcomes among PAC/PACG patients, PACS patients, and a control group for the first time. A negative correlation between AMP and refractive error was detected. Furthermore, the results indicated that patients diagnosed with PACS and PAC/PACG had significantly greater AF than those in the control group. These findings imply that the pathogenesis and progression of PACD may be linked to accommodative functions. The dynamic measurement of accommodative function could have potential clinical implications for the diagnosis and management of PACD. Future research should investigate the potential relationship between accommodation and anterior chamber depth, which may provide further insights into the pathogenesis of PACG.

## Conflicts of Interest

The authors declare no conflicts of interest.

## Data Availability

The data that support the findings of this study are available on request from the corresponding author. The data are not publicly available due to privacy or ethical restrictions.
